# Improvement in Motor and Walking Capacity during Multisegmental Transcutaneous Spinal Stimulation in Individuals with Incomplete Spinal Cord Injury

**DOI:** 10.3390/ijms25084480

**Published:** 2024-04-19

**Authors:** Hatice Kumru, Aina Ros-Alsina, Loreto García Alén, Joan Vidal, Yury Gerasimenko, Agusti Hernandez, Mark Wrigth

**Affiliations:** 1 Fundación Institut Guttmann, Institut Universitari de NeurorehabilitacióAdscrit a la UAB, 08916 Badalona, Spain; superaina11@gmail.com (A.R.-A.); lgarcia@guttmann.com (L.G.A.); jvidal@guttmann.com (J.V.); ahernandez@guttmann.com (A.H.); mawright@guttmann.com (M.W.); 2 Universitat Autònoma de Barcelona, 08193 Barcelona, Spain; 3 Fundació Institut d’Investigació en Ciències de la Salut Germans Trias i Pujol, 08916 Badalona, Spain; 4 Pavlov Institute of Physiology, St. Petersburg 199034, Russia; yuryg@ucla.edu; 5 Department of Physiology and Biophysics, University of Louisville, Louisville, KY 40292, USA

**Keywords:** transcutaneous spinal cord stimulation, multiple segmental stimulation, incomplete spinal cord injury, gait, muscle strength, spinal cord excitability

## Abstract

Transcutaneous multisegmental spinal cord stimulation (tSCS) has shown superior efficacy in modulating spinal locomotor circuits compared to single-site stimulation in individuals with spinal cord injury (SCI). Building on these findings, we hypothesized that administering a single session of tSCS at multiple spinal segments may yield greater enhancements in muscle strength and gait function during stimulation compared to tSCS at only one or two segments. In our study, tSCS was applied at single segments (C5, L1, and Coc1), two segments (C5-L1, C5-Coc1, and L1-Coc1), or multisegments (C5-L1-Coc1) in a randomized order. We evaluated the 6-m walking test (6MWT) and maximum voluntary contraction (MVC) and assessed the Hmax/Mmax ratio during stimulation in ten individuals with incomplete motor SCI. Our findings indicate that multisegmental tSCS improved walking time and reduced spinal cord excitability, as measured by the Hmax/Mmax ratio, similar to some single or two-site tSCS interventions. However, only multisegmental tSCS resulted in increased tibialis anterior (TA) muscle strength. These results suggest that multisegmental tSCS holds promise for enhancing walking capacity, increasing muscle strength, and altering spinal cord excitability in individuals with incomplete SCI.

## 1. Introduction

Spinal cord injury (SCI) is a devastating medical condition that can lead to a wide range of physical disabilities, including loss of motor function below the level of injury. Recovery from walking is one of the main goals of patients after spinal cord injury, and it occurs first, at least in patients with incomplete injuries [1,2,3,4]. On the other hand, an increase in the number of subjects with incomplete SCIs with chances of walking recovery has been reported [1,2,3,4]. Therefore, gait recovery is the goal of various pharmacological and rehabilitation approaches [5,6,7].

Gait function after SCI requires a comprehensive approach that includes medical management, physical therapy, assistive technologies, and psychosocial support. Although significant obstacles remain, the integration of research and technological innovations has the potential to improve gait function and overall quality of life for subjects living with SCI. Non-invasive rehabilitation strategies that restore or improve stepping ability have attracted substantial interest from the clinical and research communities [8].

Spinal cord stimulation (SCS) has emerged as a promising therapeutic approach for improving motor function in individuals with SCI, and transcutaneous SCS (tSCS) involves the delivery of electrical impulses to the spinal cord via transcutaneous electrodes [9,10,11,12,13,14,15,16,17,18]. Compared with single-site stimulation, transcutaneous multisegmental SCS modulated spinal locomotor circuits and facilitated locomotion more effectively in healthy individuals [19]. The use of a three-channel stimulation device permitting independent modulation via three different spinal locations at the C5, T11, and L1 vertebrae in healthy individuals induced robust oscillatory and coordinated stepping movements, and these movements were much greater than those resulting from stimulation at T11 alone [19]. In five SCI individuals, tSCS applied T11 or over coccyx 1 (Coc1) and their combination (T11 + Coc1), and rhythmic leg movements and corresponding EMG activity in leg muscles were generated during stimulation when the legs were placed in a gravity-neutral position [20]. On the other hand, a synergistic effect was reported when tSCS was applied at the coccyx (Coc1) and/or at T11 in combination with exoskeleton-assisted therapy in one individual with SCI [11].

The potential of tSCS at the cervical level to provide opportunities for remote neuromodulation has also been previously reported [21,22,23,24]. It can increase cortical excitability, affecting the responsiveness of the cerebral cortex [21,22], or it can also modulate the spinal cord reflex in the lower limb in healthy [23]. The combined transcutaneous cervical and epidural lumbar stimulation has demonstrated an enhancement in voluntary control of stepping, particularly in individuals with chronic motor complete paralysis [24].

Taken together, our hypothesis was that multisegmental tSCS (C5, L1, and Coc1) could induce neuromodulation more effectively than one or two-segmental tSCS during stimulation in a single gait training session, while the aim focused on evaluating walking time, muscle strength, and spinal cord excitability during tSCS.

## 2. Results

**Figure 1:**
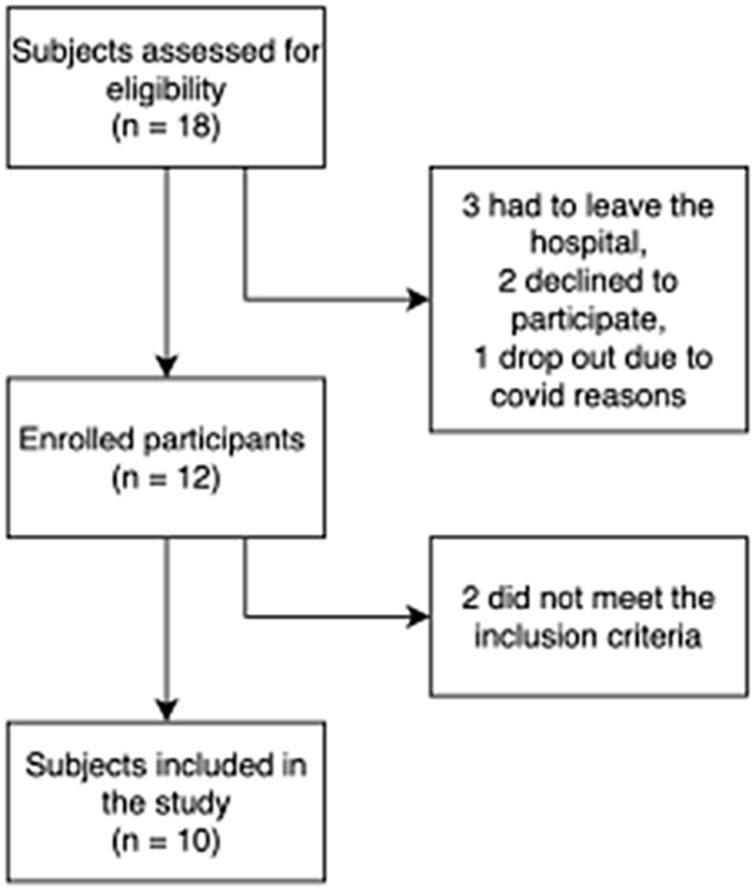
Flow diagram.

Eighteen subjects with SCI were selected for the study, but three had to be discharged from the hospital, two declined to participate, and one left the hospital because of positive COVID-19. Only twelve subjects agreed to participate, and two did not fulfill the inclusion criteria (one could not achieve a 6MWT, and the other had severe spasticity) (Figure 1). After providing written informed consent, ten subjects with SCI participated in the study. The mean age was 43.2 ± 11.5 years (range 26–61 years), with nine males and one female (Table 1).

All the subjects were able to complete the experiment without any complications.

All subjects had cervical SCI and American Spinal Injury Association (ASIA) Impairment Scale grade D (AIS-D). The mean time since SCI was 13.5 ± 20.6 months. The total motor score was 82.5 ± 11.4 and lower extremity motor score 40.8 ± 7.6. The clinical and demographic characteristics of all the subjects with SCI are given in Table 1.

The mean tolerated intensity of tSCS at C5 was 40.5 ± 11.5 mA, that at L1 was 47.9 ± 9.0 mA, and that at Coc1 was 56.4 ± 9.8 mA. The intensities used for each subject and each site of stimulation is shown in Table 1.

### 2.1. 6 Meter Walking Test (6MWT)

**Figure 2:**
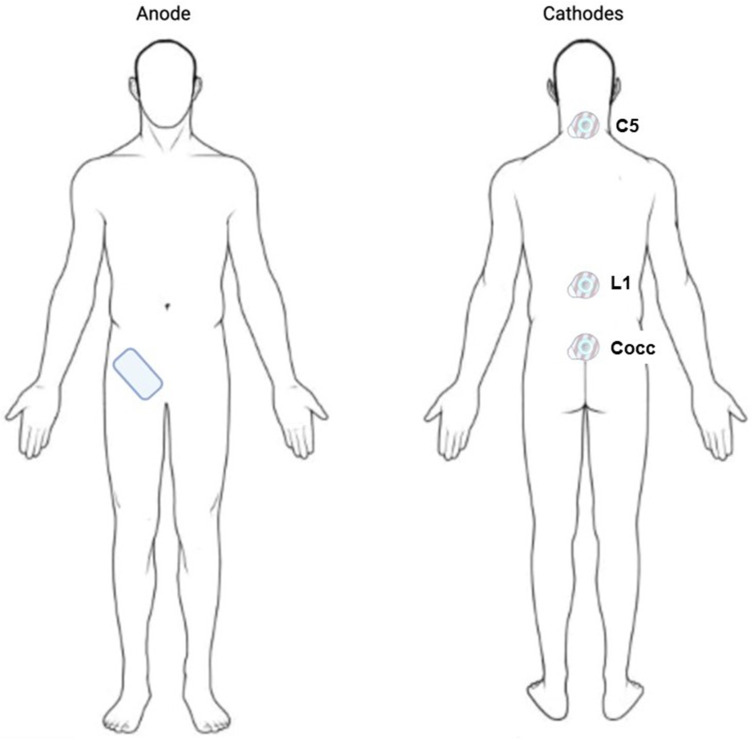
tSCS electrode placements. C—cervical; L—lumbar; Cocc—coccyx.

**Figure 3:**
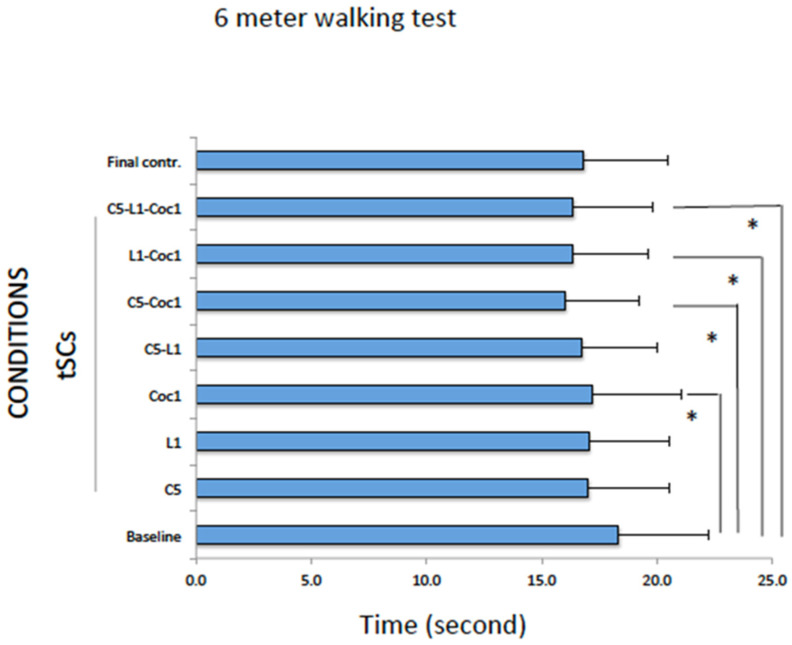
Six-meter walking test at baseline, during tSCS at different segments, and final control condition. The data were expressed as mean and standard error. * *p* ≤ 0.05 with respect to the baseline condition.

In the 6MWT, compared to the baseline condition, a significant improvement was observed during tSCS when it was applied at Coc1 (*p* = 0.047), C5-Coc1 (*p* = 0.013), L1-Coc1 (*p* = 0.013), or a combination of three segments (multisegmental tSCS; Figure 2) of C5-L1-Coc1 (*p* = 0.017) (Table 2, Figure 3).

There were no significant differences between the absolute value of tSCS conditions and of the final control (Friedman test, *p* = 0.28).

### 2.2. Maximum Voluntary Contraction of the Quadriceps Muscle (MVC-QM)

There were no significant changes in the MVC-QM between baseline, tSCS in different conditions, or the final control (Wilcoxon test, *p* > 0.05 for all conditions compared to the baseline condition) (Table 3).

There were no significant differences between the absolute value during tSCS conditions and the final control (Friedman test, *p* = 0.603).

### 2.3. Maximum Voluntary Contraction of the TA Muscle (MVC-TA)

**Figure 4:**
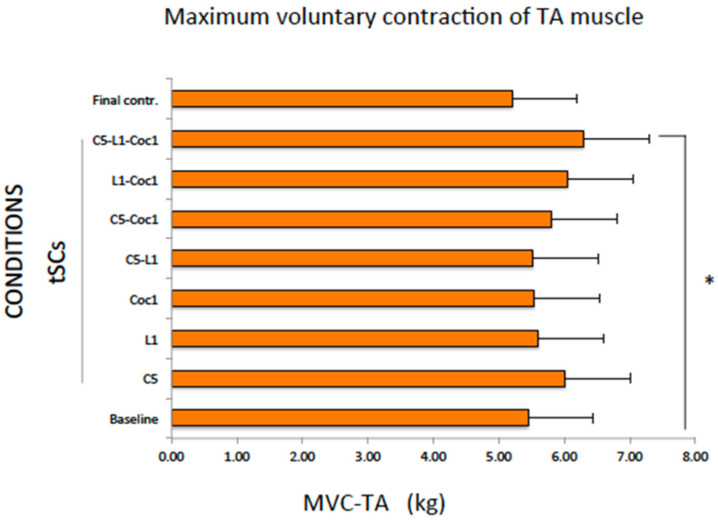
Maximum voluntary contraction of the tibial anterior muscle (kg). Data were expressed as mean and standard error. * *p* < 0.05 compared to the baseline condition (Wilcoxon test).

There was a significant increase in the maximum muscle strength of TA during multisegmental tSCS (C5-L1-Coc1) relative to baseline (Wilcoxon test, *p* = 0.037). The other conditions did not change significantly the MVC-TA (*p* > 0.05 for all comparisons with the baseline condition) (Table 4, Figure 4).

There were no significant differences between the absolute value of tSCS conditions and the final control (Friedman test, *p* = 0.439).

### 2.4. Hmax/Mmax

**Figure 5:**
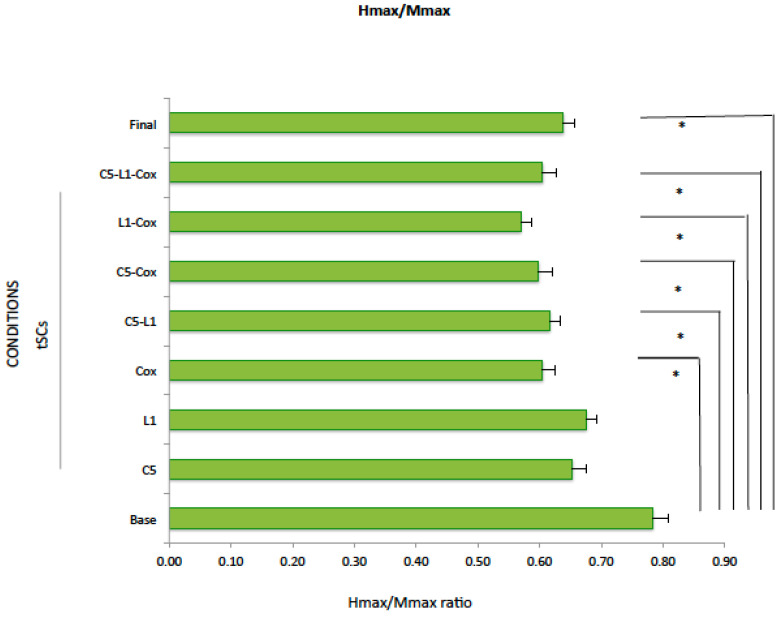
Hmax/Mmax ratio. Data were expressed as mean and standard error. * *p* < 0.05 compared to the baseline condition (Student’s *t*-test).

Despite the significant reduction in Hmax when tSCS was applied at Coc1, C5-L2, C5-Coc1, at multisegment tSCS, and under the final conditions (*p* < 0.05), there were no changes in Mmax (*p* > 0.05), except for tSCS at L1-Coc1, which increased significantly Mmax (*p* = 0.015) (Table 5A). Although Hmax/Mmax did not change when tSCS was applied at C5 (*p* = 0.09) or at L1 (*p* = 0.16), it decreased significantly when tSCS was applied at Coc1 (*p* = 0.018), C5-L1 (*p* = 0.043), C5-Coc1 (*p* = 0.008), L1-Coc1 (*p* = 0.014), or multisegmental tSCS (C5-L1-Coc1) (*p* = 0.014) with respect to the baseline conditions (Table 5B, Figure 5). The reduction in the Hmax/Mmax ratio was also significant during the final condition (*p* = 0.028).

There were no significant differences between the absolute value of tSCS conditions and the final control (Friedman test, *p* = 0.33).

## 3. Discussion

Our study revealed that tSCS at the multisegmental level (C5-L1-Coc1) improved walking time, as did tSCS at single or two segments (Coc1, C5-Coc1, and L1-Coc1). Additionally, only multisegmental tSCSs showed a significant voluntary increase in TA muscle strength. On the other hand, spinal cord excitability, as measured by the Hmax/Max ratio, significantly decreased following tSCS applied at one or two segments (at the Coc1, C5-L1, C5-Coc1, and L1-Coc1 segments) and at the multisegmental level as well.

The main mechanism of tSCS involves the non-invasive activation of neural networks within the spinal cord, potentially through the recruitment of afferent fibers to the posterior root [25,26]. Studies have demonstrated that tSCS applied at multiple spinal levels can induce robust oscillatory and coordinated stepping movements in healthy individuals [19] and generate rhythmic leg movements in individuals with SCI [20]. The combined transcutaneous cervical and epidural lumbar stimulation has demonstrated an enhancement in voluntary control of stepping, particularly in individuals with chronic motor complete paralysis [24]. On the other hand, a synergistic effect was reported when tSCS was applied at the coccyx (Coc1) and/or at T11 in combination with exoskeleton-assisted therapy in one individual with SCI [11]. The lumbosacral cord possesses rhythmogenic properties, with the rostral lumbar and sacral cords being more robust in generating motor output in animals [27,28]. The uniqueness of the lumbar cord is most likely attributable to its greater potential to generate a bursting rhythm and pattern of movement [27,28]. The sacral cord, in contrast, maintains its rhythmogenic capacity by direct activation of afferent fibers and motor axons due to the common course of ascending afferent fibers (nerve roots) around sacral segments. Additionally, ascending propriospinal circuits within the sacral cord terminate and have an excitatory effect on rostral lumbar locomotor networks [29]. We suggest that the potential significance of the interactions of this input between the lumbar and sacral neuronal circuitries improves walking capacity, and multisegment tSCS strategies that adopt spatiotemporal neuromodulation of the cervical, lumbar, and sacral cords lead to more meaningful functional motor outcomes. Additionally, tSCS at the cervical level increases cortical excitability and modulates spinal cord reflexes in the lower limb, suggesting potential opportunities for remote neuromodulation [21,22,23,24]. It has been suggested that tSCS above the site of injury at the cervical level may regulate the brain–spinal connectome and reactivate dormant descending systems [20]. Additionally, stimulation at the lumbar segment L1 has been observed to result in high current density concentrations along the cauda equina. Furthermore, sacral-coccygeal (Coc1) stimulation predominantly affects a subset of these spinal roots (S2–S4) [20]. Here, we hypothesize that a multi-site stimulation strategy targeting multiple functional spinal areas will synergistically activate spinal locomotor-related systems and assist in the recovery of motor and locomotor function. Motor and walking capacity improved because tSCS at the lumbar activates locomotor-related neural network and coccygeal level modulates of the spinal network, facilitating motor output in the lower limbs. Additional tSCS at the cervical level regulates the brain–spinal connectome and reactivates dormant descending systems. All three inputs act synergistically and effectively regulate locomotor behavior in SCI persons.

According to this study, spinal cord excitability, as measured by the Hmax/Max ratio, significantly decreased during tSCS applied at one or two segments and with multisegmental tSCS, including in the final condition. By targeting specific neural pathways, the SCS can inhibit hyperexcitable spinal reflexes [30,31,32], including the H-reflex, thereby reducing spasticity and improving motor control [14]. The previous literature suggests that spinal cord stimulation (SCS) has the potential to engage local inhibitory spinal circuits [31,32,33,34]. By stimulating afferent fibers, the SCS may trigger the release of inhibitory neurotransmitters in both humans and animals, likely by enhancing pre- and postsynaptic spinal inhibitory mechanisms [14,33,34]. The reduction in spinal cord excitability by tSCS could suggest a potential application for the normalization of spinal reflex excitability after SCI [30,31,32,33,34].

The sustained and significant alterations in spinal cord excitability, as evidenced by the Hmax/Mmax ratio during the final condition, suggest that this neuronal response may exhibit greater durability in response to non-invasive neuromodulation techniques compared to changes in walking capacity and muscle strength. This observation raises the possibility that the neural circuitry underlying the Hmax/Mmax response is distinct and more receptive to inducing enduring alterations than the circuits governing walking ability and motor strength. While these findings have significant implications for the long-term effectiveness of neuromodulation in modulating neural circuits, it remains unclear whether they directly translate to enhanced motor function in individuals with spinal cord injury. Further research is warranted to elucidate the specific mechanisms underlying these effects and their impact on motor function outcomes.

Indeed, our findings provide compelling evidence that multisegmental tSCS activates various locomotor-related spinal neural networks synergistically, leading to improvements in muscle force, walking time, and reduction in spinal cord excitability. This suggests promising potential for multisegmental tSCS in enhancing gait function and reducing spasticity following SCI, especially when combined with repeated gait therapy sessions. Such conclusions underscore the clinical relevance and potential applications of multisegmental tSCS as a valuable tool in rehabilitation interventions.

As a limitation, (i) the study had a small sample size, which may limit the generalizability of the findings. The tSCS conditions were performed randomly to determine walking ability and muscle strength, but we cannot exclude the overlap effect of the interaction between each condition that was performed accordingly. However, no significant effect was observed in the final conditions, such as walking time and muscle strength, which may exclude this possible effect. (ii) The duration of stimulation was relatively short before each assessment, which may have limited the effectiveness of each condition. (iii) The absence of a control group was one of the limitations of the study. Additionally, implementing control stimulation was challenging due to the high intensity of tSCS, which was determined based on the perception threshold. (iv) There was variability in the level and time since injury among individuals; however, all comparisons were made with their respective baseline and final control conditions. Despite this approach, it is important to acknowledge this variability as a potential limitation of the study, as it might have influenced the outcomes.

## 4. Conclusion

Our results suggest that multisegmental tSCS has potential benefits in improving gait function and increasing muscle strength and changing spinal cord excitability in the lower extremity in individuals with incomplete SCI more effectively than single or two-segmental tSCS during stimulation in a single gait training session.

However, further research and longitudinal studies are necessary to fully elucidate its effects on muscle strength, gait function, and spinal cord excitability. Optimizing stimulation parameters and duration will be crucial for enhancing and sustaining the effectiveness of this intervention.

## 5. Methods

The inclusion criteria were as follows: (i) male or female more than 18 years old; (ii) had stable traumatic or traumatic incomplete motor cervical or thoracic SCI; (iii) had an SCI for more than 6 months; (iv) had an American Spinal Injury Association Impairment Scale (AIS)-D score [35] of at least 6 m with or without technical help; and (v) had the capacity to reduce the nature of the study and signed informed consent.

The exclusion criteria were (i) unstable medical conditions (cancer, acute infections, etc.); (ii) severe spasticity (≥3 score on the modified Ashworth scale (MAS)); (iii) peripheral nerve affectation; (iv) ulcers on the electrode applied area; and (v) intolerance of tSCS.

The protocol was approved by the Ethics Committee of the ‘Unió Catalana d’Hospitals’ and was carried out in accordance with the standards of the Declaration of Helsinki. Informed consent was obtained from all individuals involved in the study.

### 5.1. Clinical Assessments

The AIS was used to evaluate clinical motor and sensory deficits according to the American Spinal Injury Association (ASIA) Impairment Scale (AIS) [35]. AIS-A indicates complete sensory and motor SCI; B indicates incomplete sensory and complete motor SCI; C and D indicate incomplete sensory and motor SCI; and AIS-E indicates normal sensory and motor function.

### 5.2. Experimental Setup

For gait function, the 6MWT was performed. The 6MWT was performed twice, once walking in one direction of the walkway and the other in reverse, and then it was repeated under tSCS conditions, which consisted of seven different combinations applied to different segments of the spinal cord in randomized order and then at the final control without any stimulation.

Maximum voluntary contraction (MVC) was performed to measure the force of two muscles, the quadriceps (QM) and the tibialis anterior (TA) muscles, which are the two main muscles involved in gait. MVC-QM was realized when the subjects were in a sitting position with the feet lightly touching the ground and the knees at a 90-degree angle. A dynamometer was positioned parallel to the ground and tied to the ankle of the more affected leg. If the legs were similarly affected, the right ankle was tied. The subjects received the imperative signal to begin realizing MVC-QM with electrical stimulation applied to the wrist with electrical stimulation applied to the wrist at the perception threshold generated by the EMG machine (Medelec Synergy, Cardinal Health, Surrey, UK). The subjects were instructed to contract the muscle by extending the knee, trying to reach their maximum, and maintaining force for 4 s.

MVC-TA was performed while the subjects were in a semi-sitting position in bed with the back reclined 45 degrees and the knee flexed 30 degrees and supported underneath by a special pillow. The dynamometer, which was tied at the level of the metatarsal-phalangeal joint, was positioned parallel to the ground, in line with the leg of the subject. In both tests, the maximum and force maintained for 4 s were accepted by the dynamometer.

First, individuals with SCI performed MVC-QM in the sitting position for all conditions, and then, they were given time to rest before performing MVC-TA in the lying position.

After performing MVC-TA under each condition, we recorded Hmax and Mmax to calculate the Hmax/Mmax ratio as a measure of changes in spinal cord excitability. The H reflex is the tool most commonly used to investigate modulations in spinal excitability [36,37,38]. Surface electrodes (Technomed disposable adhesive 4-disk electrode, Netherlands) were placed over the soleus muscle, and the electrical stimulator was fixed at the optimal point to record the H reflex in the popliteal fossa.

The 6MWT and MVC for each muscle and each condition were repeated twice. Hmax/Mmax was recorded once. The 6MWT and MVC were performed on different days to avoid fatigue and overlapping assessments.

### 5.3. Study Conditions

This study consisted of (i) baseline conditions and (ii) tSCS conditions, which consisted of seven different combinations applied to different segments of the spinal cord in the following randomized order: C5, L1, Coc1, C5+L1, C5+Coc1, L1+Coc1, C5+L1+Coc1, and (iii) final conditions. Individuals with SCI completed all clinical assessments after the last tSCS session. We included the final control condition to ensure the reliability of our findings.

Baseline, tSCS, and final control conditions were used for three assessments: the 6MWT, MVC-QM, MVC-TA, and Hmax/Mmax ratio.

The interventions aimed to assess the effect of tSCS at different stimulation locations on walking and MVC of different muscles and spinal cord excitably measured by the Hmax/Mmax ratio, (i) stimulation at each point of stimulation (C5, L1, and Coc1); (ii) different combinations of these two points (C5+L1, C5+Coc1, and L1+Coc1); and (iii) combinations of all three points (C5+L1+Coc1). The order of stimulation conditions was randomized to minimize bias. The purpose of these assessments was to evaluate the effects of tSCS on walking performance, muscle strength, and spinal cord excitability.

In each tSCS condition, stimulation was on for 2 min to ensure that participants received stimulation at the specified locations before any test.

Between each condition, participants rested for 1 to 2 min to minimize fatigue and allow recovery for the next condition.

The 6MWT and MVC were repeated twice for each condition (baseline, tSCS in random order at C5, L1, Coc1, C5+L1, C5+Coc1, L1+Coc1, C5+L1+Coc1, and other conditions), but Hmax and Mmax were recorded only once for each condition.

### 5.4. Stimulation

The tSCS was delivered through circular hydrogel adhesive electrodes (2 cm diameter, Axion GmbH, Leonberg, Germany) placed along the midline over spinous processes C5, L1, and Coc1 (midline on the sacral-coccygeal region) (Figure 2). The tSCS was delivered using biphasic rectangular 1 ms pulses at a frequency of 30 Hz, with each pulse filled with a carrier frequency of 10 kHz. For the stimulation, three channels of a five-channel current-controlled stimulator of Biostim-5 stimulator (Cosyma Inc., Moscow, Russia) were used. Each channel was set up independently.

First, channel stimulation was delivered by a cathode at the C5 level; second, channel stimulation by a cathode at the L1 level; and third, channel stimulation by a cathode at the Coc1 level (Figure 2). One anode was placed at the right iliac crest (Figure 2). The appropriate stimulation intensity was identified for each subject as the highest intensity tolerated at each stimulation site (Table 1), and it was applied one day before the experimental conditions.

## 6. Data Analysis and Statistics

For Hmax and Mmax, we measured the peak-to-peak amplitude in the largest response and then calculated the Hmax/Mmax ratio.

All the data are expressed as the means and standard deviations or standard errors. The means of the 6MWT, the MVC-QM, and the MVC-TA were calculated from two trials for each individual, and the means and standard errors were calculated for the baseline, for all tSCS conditions, and for final control.

The Shapiro–Wilk test was used to evaluate whether the data were normally distributed. The data were not normally distributed, as were the 6MWT, MVC-QM, and MVC-TA data; the Wilcoxon test was used to compare the effects of tSCS under different conditions and the final control in comparison to baseline conditions. For normally distributed data such as Hmax, Mmax, and Hmax/Mmax, we used paired *t*-tests.

For comparison between the tSCS conditions and the final control, we calculated the absolute value after the tSCSs and final control conditions. For multiple comparisons, the Friedman test was used. When the differences were significant, the Wilcoxon test was used for post hoc analysis. The alpha level was set at 0.05 for all comparisons.

## Figures and Tables

**Table 1:** Demographic data and stimulation parameters of the SCI subjects.

Demographic Data									Intensity of tSCS		
Subject	Age	Gender	AIS	NLI	Etiology	Time since SCI (Months)	Total Motor Score	LEMS	C5 (mA)	L1 (mA)	Coc1 (mA)
tSCS 1	32	M	D	C4	T	6	90	45	25	42	56
tSCS 2	28	M	D	C8	T	6	89	39	28	58	60
tSCS 3	48	M	D	C4	NT	6	96	49	25	55	53
tSCS 4	55	M	D	C3	T	6	88	46	40	50	40
tSCS 5	61	F	D	C4	T	72	81	38	44	50	62
tSCS 6	42	M	D	C5	T	6	81	43	50	44	62
tSCS 7	47	M	D	C6	T	11	57	26	40	49	60
tSCS 8	26	M	D	C7	T	6	90	45	46	30	46
tSCS 9	50	M	D	C6	T	8	70	30	47	41	50
tSCS 10	43	M	D	C3	T	8	83	47	60	60	75

AIS—American Spinal Injury Association Impairment Scale; M—male; F—female; T—traumatic; NT—non-traumatic; NLI—neurological level of injury; LEMS—lower extremity motor score; C—cervical; L—lumbar; Coc1—coccyx-1.

**Table 2:** 6MWT (seconds) of each individual.

Subject	Baseline	C5	L1	Coc1	C5-L1	C5-Coc1	L1-Coc1	C5-L1-Coc1	Final Control
tSCS 1	6.09	6.88	6.67	6.75	6.51	6.19	6.58	5.94	5.99
tSCS 2	7.80	8.80	10.43	8.09	8.59	8.03	8.57	7.78	8.19
tSCS 3	13.36	11.22	11.13	11.29	11.06	10.81	10.75	11.11	11.16
tSCS 4	7.07	5.97	6.58	6.16	6.39	6.08	6.05	5.99	5.81
tSCS 5	23.81	23.82	24.40	21.38	23.85	23.34	23.02	24.14	23.20
tSCS 6	13.48	11.48	9.76	9.45	13.83	12.27	12.11	11.52	9.86
tSCS 7	39.70	37.77	36.41	37.58	30.99	30.67	34.60	29.64	32.50
tSCS 8	9.43	8.89	8.73	8.71	8.54	8.62	8.47	8.29	8.44
tSCS 9	36.02	29.19	30.58	36.07	31.13	31.59	29.24	35.89	34.76
tSCS 10	26.51	26.15	25.66	26.24	26.58	22.63	23.92	23.40	28.36
Mean	18.33	17.02	17.03	17.17 *	16.74	16.02 *	16.33 *	16.37*	16.82
SE	3.92	3.55	3.50	3.88	3.24	3.18	3.29	3.45	3.66

SE: standard error; * *p* < 0.05 compared to the baseline condition (Wilcoxon test).

**Table 3:** Maximum voluntary contraction of the quadriceps muscle (kg).

Subjects	Baseline	C5	L1	Coc1	C5-L1	C5-Coc1	L1-Coc1	C5-L1-Coc1	Final Control
tSCS 1	15.59	13.56	12.93	13.18	12.49	12.15	14.78	12.79	12.39
tSCS 2	7.30	6.81	8.49	9.59	8.84	10.06	11.48	10.26	8.74
tSCS 3	11.80	26.58	23.49	21.80	19.24	20.81	14.44	24.43	22.20
tSCS 4	26.47	30.00	28.45	27.25	29.34	29.72	27.20	28.67	28.85
tSCS 5	6.80	8.58	6.27	6.20	5.80	6.91	5.37	6.65	8.05
tSCS 6	17.07	16.41	15.94	16.90	15.88	16.63	16.00	16.60	17.27
tSCS 7	18.41	14.69	16.23	15.08	16.65	16.56	15.47	16.50	17.25
tSCS 8	25.92	29.07	30.00	26.89	28.14	30.00	26.67	29.50	27.42
tSCS 9	14.30	10.73	13.80	12.06	14.90	11.89	12.66	11.25	11.18
tSCS 10	19.41	18.47	19.69	19.27	19.21	19.30	17.74	19.59	20.15
Mean	16.30	17.49	17.53	16.82	17.05	17.40	16.18	17.62	17.35
SE	2.13	2.66	2.50	2.22	2.37	2.47	2.08	2.48	2.33

There was not any significant difference in MVC-QM in any condition with respect to baseline.

**Table 4:** Maximum voluntary contraction of the tibial anterior muscle (kg).

Subject	Baseline	C5	L1	Coc1	C5-L1	C5-Coc1	L1-Coc1	C5-L1-Coc1	Final Control
tSCS 1	7.62	7.46	8.75	8.85	7.54	8.03	11.25	11.00	8.04
tSCS 2	0.64	0.79	0.68	0.80	0.07	1.03	0.54	0.56	0.74
tSCS 3	3.12	3.02	3.27	2.35	1.55	4.66	5.16	2.40	2.49
tSCS 4	13.02	13.33	13.64	13.94	13.56	13.46	11.91	15.11	13.61
tSCS 5	0.35	1.03	0.90	1.51	1.09	1.03	0.30	0.64	0.41
tSCS 6	8.44	7.90	7.43	7.29	9.96	7.23	10.22	9.41	7.39
tSCS 7	2.84	4.14	3.17	2.10	3.25	3.73	3.02	3.10	3.33
tSCS 8	7.34	11.01	7.78	8.30	8.48	8.93	8.57	9.20	7.54
tSCS 9	4.90	5.27	4.97	3.70	4.84	4.80	4.56	5.05	3.45
tSCS 10	6.13	6.09	5.35	6.46	4.81	5.09	4.94	6.40	4.96
Mean	5.44	6.00	5.59	5.53	5.51	5.80	6.05	6.29 *	5.20
SE	0.54	0.60	0.56	0.55	0.55	0.58	0.60	0.63	0.52

SE: standard error; * *p* < 0.05 compared to the baseline condition (Wilcoxon test).

**Table 5:** **A.** Hmax and Mmax (microV); **B**. Hmax/Mmax ratio.

A																		
	Hmax (microV)									Mmax (microV)								
	Base	C5	L1	Coc1	C5-L1	C5-Coc1	L1-Coc1	C5-L1-Coc1	Final	Base	C5	L1	Coc1	C5-L1	C5-Coc1	L1-Coc1	C5-L1-Coc1	Final
tSCS 1	5122	2756	3659	3476	2561	2634	4561	2659	3000	8171	8354	9085	9024	7866	8842	8964	9024	9146
tSCS 2	5000	4390	4902	4293	3122	4390	4951	4268	4073	7500	5317	7098	6634	6317	6878	6365	5713	6146
tSCS 3	4298	4634	4573	4573	3659	4146	3792	3963	3969	5603	5634	6281	5122	5366	5854	7273	6159	6780
tSCS 4	7505	7285	6688	7096	6633	6407	8358	7322	7286	11,926	11,030	11,913	13,048	13,700	10,993	13,946	13,060	12,317
tSCS 5	2524	1152	2835	1829	1402	1225	ND	1823	2414	3396	2707	3493	2878	2884	2804	ND	3231	3213
tSCS 6	5341	4341	3914	3384	4778	4701	4341	2914	4646	6103	8042	8407	8656	8887	8548	7743	8048	8657
tSCS 7	1342	1781	2049	1512	2146	1853	1537	1781	1488	3732	3659	2976	4610	2902	4488	4756	5439	2756
tSCS 8	4146	3122	3659	3512	3598	3073	2683	4171	3537	4561	6073	5915	6293	5671	6707	6342	6171	6281
tSCS 9	4634	4000	4195	3854	4073	4024	3805	3781	3902	3537	4000	4439	4390	4659	3951	4366	4048	3756
tSCS 10	5463	5854	5390	4781	5024	6037	5659	5407	5549	5829	6342	6415	6098	5585	6890	6171	5732	7073
Mean	4537.5	3931.5	4186.4	3831.0 *	3699.6 *	3849.0 *	4409.7 *	3808.9	3986.4 *	6035.8	6115.8	6602.2	6675.3	6383.7	6595.5	7325.1 *	6662.5	6612.5
SE	167.8	183.2	130.8	156.8	152.7	168.0	213.2	168.1	162.2	263.2	249.3	270.8	292.2	318.9	246.6	317.4	280.5	295.0
**B**																		
		**Hmax/Mmax**																
**Subject**		**Baseline**		**C5**		**L1**	**Coc1**		**C5-L1**		**C5-Coc1**		**L1-Coc1**		**C5-L1-Coc1**		**Final Control**	
tSCS 1		0.63		0.33		0.40	0.39		0.33		0.30		0.51		0.29		0.33	
tSCS 2		0.67		0.83		0.69	0.65		0.49		0.64		0.78		0.74		0.66	
tSCS 3		0.77		0.82		0.73	0.89		0.68		0.71		0.52		0.64		0.59	
tSCS 4		0.63		0.66		0.56	0.54		0.48		0.58		0.56		0.56		0.59	
tSCS 5		0.74		0.43		0.81	0.64		0.49		0.44		ND		0.56		0.75	
tSCS 6		0.88		0.54		0.47	0.39		0.54		0.55		0.56		0.36		0.54	
tSCS 7		0.36		0.49		0.69	0.33		0.74		0.41		0.32		0.33		0.54	
tSCS 8		0.91		0.51		0.62	0.56		0.63		0.46		0.42		0.68		0.56	
tSCS 9		1.31		1.00		0.95	0.88		0.87		1.02		0.87		0.94		1.04	
tSCS 10		0.94		0.92		0.84	0.78		0.90		0.88		0.54		1.00		0.78	
Mean		0.78		0.65		0.68	0.60 *		0.62 *		0.60 *		0.56 *		0.60 *		0.64 *	
SE		0.08		0.03		0.02	0.02		0.02		0.02		0.02		0.02		0.02	

SE—standard error. * *p* < 0.05 compared to the baseline condition (Student’s *t*-test). ND—not done.

## Data Availability

All the data and materials can be found at Institut Guttmann.
